# Are there clinical and sociodemographic differences between bipolar i and ii disorders?

**DOI:** 10.1192/j.eurpsy.2022.1061

**Published:** 2022-09-01

**Authors:** F. Piazza, A. Rodríguez Rey, L. Montejo, B. Solé, A. Martínez-Arán

**Affiliations:** 1 Clinic Hospital, Psychiatry And Psychology, Barcelona, Spain; 2 Hospital Clinic of Barcelona, Department Of Psychiatry And Psychology, Clinical Institute Of Neuroscience, Barcelona, Spain; 3 Clinical Institute of Neuroscience, Bipolar And Depressive Disorders Unit, Hospital Clinic Of Barcelona, Idibaps, Cibersam Spain, Barcelona, Spain

**Keywords:** bipolar disorder add clinical add SOCIODEMOGRAPHIC add DIFFERENCES

## Abstract

**Introduction:**

Bipolar disorder is a serious mental disorder. Although bipolar disorder I (BDI) might seem to have a more complex evolution and severe prognosis than bipolar disorder II (BDII) because of cross-sectional symptom severity, BDII has a high episode frequency, high rates of psychiatric comorbidities and recurrent suicidal behaviours that impair functioning and quality of life.

**Objectives:**

To explore whether there are differences between patients with BDI and BDII concerning sociodemographic and clinical variables of interest.

**Methods:**

A sample of 407 euthymic patients with bipolar disorder (307 BDI and 100 BDII) being age 18 or older was recruited from the Bipolar and Depressive Disorders Unit of the Hospital Clinic of Barcelona. Sociodemographic and clinical variables were collected through the administration of semi-structured interview and clinical scales. Differences between groups in these variables were analysed using the Mann-Whitney U and Chi-square tests, as appropriate. The level of significance was set at p <0.05.

**Results:**

We found statistically significant differences between both groups. Patients with BD II were older (p<0.001), presented a longer illness duration (p=0,001) and a greater subsyndromal depressive symptomatology (p=0,010). Patients with BDI had a higher number of previous hospitalizations (p<0,001) and higher rates of psychotic symptoms (p<0,001) even during the first episode (p<0,001).

**Conclusions:**

Our data suggests that clinical differences exist between both bipolar subtypes. The episodes may be more serious, with a greater presence of a history of psychosis, and require more hospitalizations in BDI patients. In the BDII group, persistent subsyndromal symptoms may predominate, especially of the depressive pole.

**Disclosure:**

No significant relationships.

